# *PTPRT* and *PTPRD* Deleterious Mutations and Deletion Predict Bevacizumab Resistance in Metastatic Colorectal Cancer Patients

**DOI:** 10.3390/cancers10090314

**Published:** 2018-09-06

**Authors:** Hung-Chih Hsu, Nina Lapke, Shu-Jen Chen, Yen-Jung Lu, Ren-Shiang Jhou, Chien-Yuh Yeh, Wen-Sy Tsai, Hsin-Yuan Hung, Jason Chia-Hsun Hsieh, Tsai-Sheng Yang, Tan Kien Thiam, Jeng-Fu You

**Affiliations:** 1 Division of Hematology/Oncology, Chang Gung Memorial Hospital at Linkou, Taoyuan City 333, Taiwan; dannyhsuyoyo@gmail.com (H.-C.H.); wisdom5000@gmail.com (J.C.-H.H.); a481124@cgmh.org.tw (T.-S.Y.); 2 College of Medicine, Chang Gung University, Taoyuan City 33302, Taiwan; chnyuh@gmail.com (C.-Y.Y.); wensyt@gmail.com (W.-S.T.); hsin3345@gmail.com (H.-Y.H.); 3 ACT Genomics, Neihu Dist., Taipei City 114, Taiwan; ninalapke@actgenomics.com (N.L.); sjchen@actgenomics.com (S.-J.C.); yjlu@actgenomics.com (Y.-J.L.); renshiangjhou@actgenomics.com (R.-S.J.); 4 Division of Colon and Rectal Surgery, Chang Gung Memorial Hospital at Linkou, Taoyuan City 333, Taiwan

**Keywords:** metastatic colorectal cancer, bevacizumab resistance, next-generation sequencing, VEGF, *PTPRT*/*PTPRD* mutation and deletion

## Abstract

Background: Bevacizumab-based regimens are used as standard treatments for colorectal cancer. Unfortunately, there are no established predictive markers for bevacizumab response. Methods: Tumor samples from 36 metastatic colorectal cancer patients treated with bevacizumab plus chemotherapy were analyzed by next-generation sequencing of all coding exons of more than 400 genes. Single gene and signaling pathway analyses were performed to correlate genomic data with response. Results: Among the genes most frequently mutated in our cohort, only mutations in *PTPRT*, a phosphatase involved in *JAK/STAT* signaling, were associated with response status, with deleterious mutations being enriched in non-responders. Pathway analysis revealed that deleterious mutations in genes of the *JAK/STAT* pathway, namely in *PTPRT* and the related gene *PTPRD*, correlated with resistance. Mutations in *RTK/PI3K/RAS*, *Wnt* and *TGFβ* pathways did not associate with response. Lack of response was observed in all patients with deleterious mutations or copy number loss of *PTPRT*/*PTPRD* (*n* = 10), compared to only 30.8% (*n* = 8) of patients without such alterations (relative risk, 3.25; 95% CI, 1.83–5.79, *p* = 0.0003). Similarly, *PTPRT*/*PTPRD* deleterious alterations were associated with shorter progression-free survival, an association that was retained in multivariate analysis (HR, 3.33; 95% CI, 1.47–7.54; *p* = 0.0038). Conclusion: Deleterious alterations in *PTPRT*/*PTPRD* are potential biomarkers for bevacizumab resistance.

## 1. Introduction

Colorectal cancer (CRC) is one of the most common cancer types worldwide [1]. It accounts for 8.5% of all tumor-related mortality, and it is the fourth most common cause of cancer death [1]. Colorectal cancer diagnosed at an early stage is associated with a good prognosis. However, 20–25% of CRC patients initially present with metastases, and approximately half of non-metastatic patients will eventually develop metastatic disease [2]. Unfortunately, metastatic CRC (mCRC) patients have a poor prognosis, and mCRC accounts for the high mortality rates associated with CRC.

Systemic chemotherapy (i.e., fluoropyrimidines, irinotecan, and oxaliplatin) is still the main treatment for mCRC. In the last decades, novel targeted agents combined with chemotherapy or used alone have largely improved therapy outcomes for mCRC patients, and some agents are already established in the clinic. These target agents include the anti-epidermal growth factor receptor (EGFR) antibodies cetuximab and panitumumab, as well as the vascular endothelial growth factor (VEGF) inhibitors bevacizumab, aflibercept, and ramucirumab [3].

Bevacizumab is a monoclonal antibody directed against vascular endothelial growth factor A (VEGF-A). By inhibiting the action of VEGF-A, bevacizumab induces the regression of tumor vasculature and prevents new blood vessels formation, hindering tumor growth [4]. In mCRC, the combination of bevacizumab with chemotherapy (5-fluorouracil/leucovorin/irinotecan or 5-fluorouracil/leucovorin) in first-line therapy has been shown to be associated with increased clinical survival benefit [4,5,6,7]. Therefore, bevacizumab in combination with standard chemotherapy is recommended as a first-line treatment for mCRC patients [8,9]. However, according to clinical practice guidelines, there are currently no predictive biomarkers to determine the use of bevacizumab in mCRC [8,9,10].

Regarding such biomarkers, recent literature indicated that the VEGF-A polymorphisms rs3025039 and rs833061 were associated with survival [11,12]. However, these results could not be confirmed in another study [13]. Sohn et al. [14] reported that a polymorphism in the vascular endothelial growth factor receptor-1 (VEGFR-1), which binds VEGF-A and other ligands, is a predictive biomarker of bevacizumab sensitivity. Furthermore, high levels of VEGF-D, which is not targeted by bevacizumab, have been associated with resistance [15]. In fact, the existence of VEGFs other than VEGF-A and the complexity of angiogenesis signaling could contribute to the challenge to identify biomarkers of response. Further, biomarker analyses have largely neglected genetic alterations in tumor cells that may influence drug sensitivity, and more comprehensive biomarker analyses are needed.

To uncover genetic biomarkers that may predict bevacizumab sensitivity or resistance, we comprehensively analyzed tumor samples of 36 mCRC patients who received bevacizumab therapy. Tumor samples were subjected to high-throughput sequencing of 409 cancer-related genes, and genetic data were correlated with clinical outcome.

## 2. Results

### 2.1. Demographic Characterization of Patients

Thirty-six mCRC patients received bevacizumab and chemotherapy combination therapy, with 88.9% (*n* = 32) of patients receiving first-line and 11.1% (*n* = 4) of patients receiving second-line treatment (Table 1). Follow-up intervals ranged from weekly to every three months. On average, the follow-up duration was 26.2 months (standard deviation: 18.7 months). Follow-up ended at the time of death or on 1 February 2018. Patients were diagnosed at a median age of 59.5 years (range: 33–87 years, standard deviation 13.3 years), and there were similar proportions of patients with colon cancer (58.3%, *n* = 21) and rectal cancer (41.7%, *n* = 15). Tumor staging was performed based on the American Joint Committee on Cancer (AJCC) 2010 guidelines.

To identify genetic biomarkers associated with bevacizumab response, patients were stratified into responders with partial remissions (50.0%, *n* = 18) and non-responders (50.0%, *n* = 18). Among non-responders, 72.2% (*n* = 13) of patients had stable disease and 27.8% (*n* = 5) had progressive disease. The disease-control rate was 86.1% (31 of 36) among all subjects. The median progression-free survival (PFS) and response duration were 9.8 months and 7.3 months, respectively. The most common metastatic site was the liver. Regarding the number of metastatic organs, there was no difference between the responder and non-responder group; 58.3% of subjects had one metastatic organ.

### 2.2. Deleterious Mutations in the Phosphatase Gene PTPRT Are Associated with Bevacizumab Response Status

**Figure 1:**
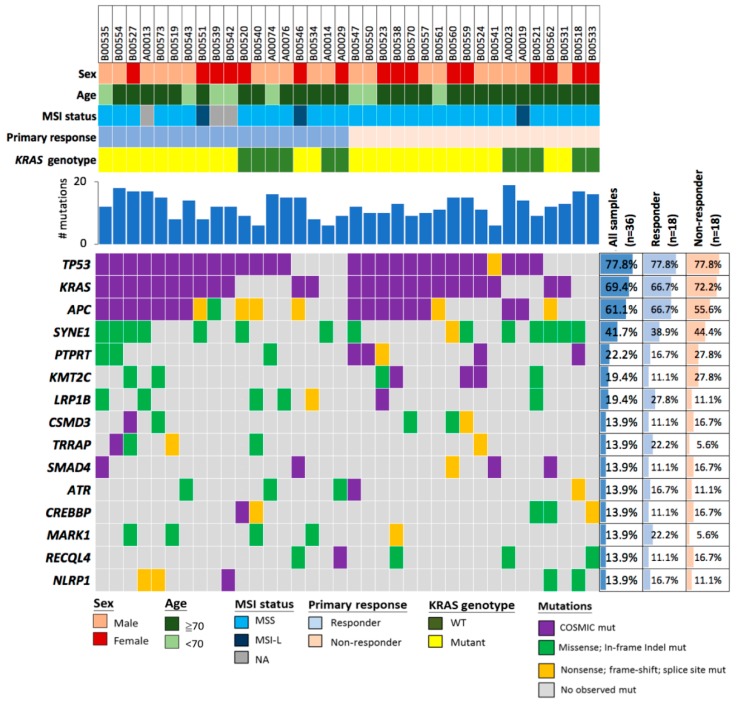
Mutations detected in tumor samples of colorectal cancer patients according to bevacizumab response status. Genes for which genetic mutations were detected in at least five study patients are depicted in an oncoprint plot and were ranked according to their observed mutation frequency in the overall study cohort. Patients were grouped according to their bevacizumab response status. Mutations were subdivided into variants with or without existing COSMIC ID. Variants without COSMIC ID were either truncating variants—including nonsense, frameshift, and splice-site variants or missense variants and in-frame indel variants. Additionally, the patient characteristics sex, age, MSI status, and total number of detected mutations are indicated for each patient.

**Figure 2:**
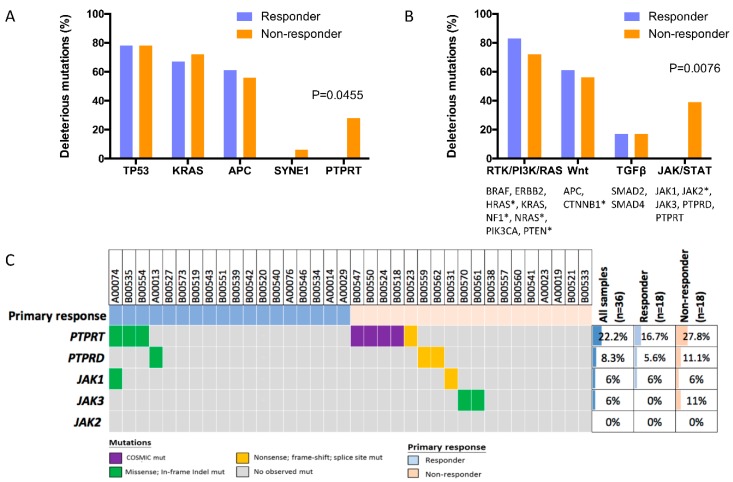
Mutations in *PTPRT*/*PTPRD*, genes of the JAK/STAT pathway, discriminate between bevacizumab responders and non-responders. Postulated deleterious mutations in single genes with high mutation frequencies in our cohort (**A**) and in multiple genes involved in important colorectal cancer (CRC) signaling pathways (**B**) were evaluated for bevacizumab responders and non-responders. Genes included in the pathway-based analysis are listed, and genes without any detected mutations in our cohort are indicated by a star. Postulated deleterious variants were variants with existing COSMIC ID or—if the analyzed genes were tumor suppressor genes—truncating variants. In our analysis, genes with detected mutations that were considered as tumor suppressors were *APC*, *PTPRD*, *PTPRT*, *SMAD2*, *SMAD4*, *SYNE1*, and *TP53*. The proportion of responders and non-responders harboring such variants is depicted. Statistical analysis was performed with the Fisher’s exact test. An oncoprint of genes involved in JAK/STAT signaling is shown (**C**).

To investigate which genes could be used to discriminate between mCRC patients with or without a response to bevacizumab treatment, we analyzed 409 cancer-related genes (see Supplementary Table S1 for a complete list of genes assayed). A total of 439 mutations (mean 12.2, range 6–19), including 409 base substitutions and 30 small indels, occurring in 185 genes were detected in this cohort. The average number of mutations was similar in tumors of responders (12.3, range 6–18) and non-responders (12.1, range 6–19) (see Supplementary Table S2 for a complete list of all detected sequence variants). The five most frequently mutated genes were *TP53*, *KRAS*, *APC*, *SYNE1*, and *PTPRT*, being mutated in 77.8% (*n* = 28), 69.4% (*n* = 25), 58.3% (*n* = 21), 41.7% (*n* = 15), and 22.2% (*n* = 8) of patients (Figure 1). We reasoned that previously observed cancer variants with existing COSMIC ID (COSMIC-recurrent mutations), as well as loss-of-function (LoF) mutations in tumor suppressor genes (TSGs), including nonsense, frameshift, and splice-site variants, would be more likely to be deleterious. In line with previous studies on CRC, mutations observed in *TP53*, *KRAS*, and *APC* were mostly such postulated deleterious variants, and had a similar frequency in both responders and non-responders (Figure 2A and Supplementary Table S3). Only one *SYNE1* variant was postulated deleterious. However, all five non-responders who harbored mutations of the TSG *PTPRT* had previously observed cancer variants or truncating variants, whereas this was not the case for any of the three responders with *PTPRT* variants. These results indicated that postulated deleterious *PTPRT* variants might discriminate between bevacizumab responders and non-responders, and deleterious *PTPRT* variants were significantly associated with lack of response (relative risk, 2.39; 95% CI, 1.58–3.61, *p* = 0.0455, Figure 2A and Supplementary Table S3). In addition to the five most frequently mutated genes, there were two genes with mutations in each 19.4% (*n* = 7) of patients, namely *KMT2C* and *LRP1B* (Figure 1). However, neither the distribution of any variants in those genes, nor the distribution of postulated deleterious variants, were significantly different between responders and non-responders (*p* > 0.05, Fisher’s exact test).

### 2.3. Deleterious Mutations in PTPRT and PTPRD, Phosphatases of the JAK/STAT Pathway, Predict Bevacizumab Response

To evaluate mutations associated with response more comprehensively and overcome the limitations of analyzing mutations in single genes, genes involved in important CRC cancer signaling pathways were grouped and analyzed together. There was no association of response status with mutations in genes of the *RTK/PI3K/RAS* pathway (*BRAF*, *ERBB2*, *HRAS*, *KRAS*, *NF1*, *NRAS*, *PIK3CA*, and *PTEN*, *p* = 0.6906), Wnt pathway (*APC* and *CTNNB1*, *p* = 1.0000), and TGFβ pathway (*SMAD2* and *SMAD4*, *p* = 1.0000) (Figure 2B and Supplementary Table S3).

However, deleterious mutations in TSGs of the *JAK/STAT* pathway (*PTPRD* and *PTPRT*) were significantly associated with bevacizumab resistance, with 38.9% (*n* = 7) of non-responding patients, but none of the responding patients harboring such mutations. The relative risk for non-response in patients with deleterious mutations in *JAK/STAT* pathway genes was 2.64 compared to the remaining study patients (95% CI, 1.66–4.20, *p* = 0.0076). Importantly, non-*PTPRT* postulated deleterious variants were only detected in the *PTPRT*-related phosphatase gene *PTPRD*, occurring in two non-responders (Figure 2C). In contrast, no oncogenic in *JAK1*-, *JAK2*- or *JAK3*-activating mutation that could result in constitutive *JAK/STAT* activation was detected in both subsets.

### 2.4. PTPRT Phosphatase Domain Missense Variants and PTPRT/PTPRD Truncating Variants Are Characteristic for Bevacizumab Non-Responders

**Figure 3:**
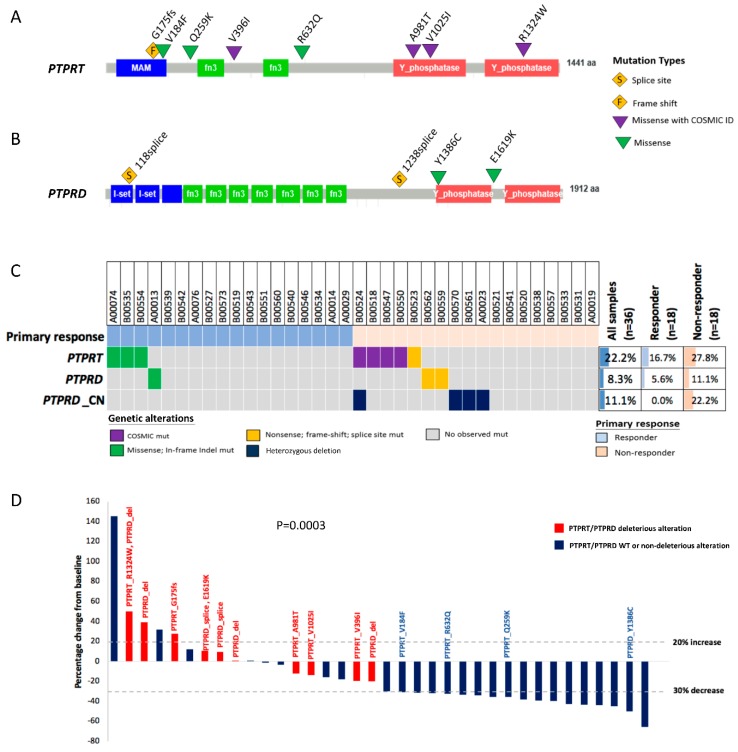
Observed *PTPRT/PTPRD* genetic alterations and bevacizumab response. Detected variants in the genes *PTPRT* (**A**) and *PTPRD* (**B**) are depicted according to their protein localization and were classified according to the observed variant type as splice-site variants, frameshift variants, and missense variants with or without COSMIC ID. A *PTPRD* missense variant (E1619K) and a *PTPRD* splice variant were observed in the same patient. *PTPRT/PTPRD* variants and *PTPRD* copy number loss are depicted in an oncoprint plot, with patients grouped according to bevacizumab response status (**C**). Maximum tumor lesion changes from baseline and *PTPRT/PTPRD* genetic alterations are displayed in a waterfall plot (**D**). The presence or absence of postulated deleterious *PTPRT/PTPRD* genetic alterations—including mutations and copy number losses—among responders and non-responders was analyzed by the Fisher’s exact test.

To evaluate the potential functional impact of *PTPRT* and *PTPRD* missense variants, we analyzed their protein domain localization. Details regarding all observed *PTPRT* and *PTPRD* variants are listed in Table 2. Three of the four *PTPRT* COSMIC-recurrent mutations observed in non-responders were located in the functionally important protein-tyrosine phosphatase domains (A981T, V1025I and R1324W), whereas the remaining variant (V396I) was located outside of functionally well-defined domains (Figure 3A). Among the three *PTPRT* missense variants without COSMIC ID observed in responders, only one variant (V184F) was located in a functional domain, the extracellular meprin-A5 antigen-PTP (MAM) domain. Two *PTPRD* missense variants were observed in each a responder and a non-responder, none of which was listed in COSMIC (Figure 3B). However, the *PTPRD* variant observed in the non-responding patient (E1619K) co-occurred with a *PTPRD* splice-site variant. The variant observed in the responding patient (Y1386C) was located in the phosphatase domain.

### 2.5. Combining Copy Number Loss and Deleterious Mutations Improves the Predictive Power of PTPRT/PTPRD for Bevacizumab Response

Since deleterious genetic alterations in TSGs are not limited to inactivation by mutations, but also by copy number loss, we analyzed whether such copy number losses in the genes *PTPRD* and *PTPRT* occurred in our study cohort (Figure 3C and Table 2). Although no copy number loss was observed for *PTPRT*, *PTPRD* heterozygous deletions occurred in four patients, in whom only one gene copy was observed in the tumor tissue. Importantly, *PTPRD* loss was only detected in bevacizumab non-responders. *PTPRD* loss co-occurred with a deleterious *PTPRT* variant (R1324W) in one patient. Otherwise, postulated deleterious *PTPRT* and *PTPRD* alterations were mutually exclusive (Figure 3C). 

Tumor change from baseline and the genetic alteration status for *PTPRT/PTPRD* are displayed in a waterfall plot (Figure 3D). In total, 55.6% (*n* = 10) of bevacizumab non-responders, but none of the responders harbored postulated deleterious *PTPRT/PTPRD* alterations (*p* = 0.0003). Therefore, the overall response rate (complete response (CR) + partial response (PR)) among patients with deleterious *PTPRT/PTPRD* alterations was 0.0% (0/10), compared to 69.2% (18/26) among patients without deleterious *PTPRT/PTPRD* alterations. The relative risk for the lack of response in patients with deleterious *PTPRT/PTPRD* alterations was significantly enhanced compared to patients without such alterations (relative risk, 3.25; 95% CI, 1.83–5.79, *p* = 0.0003).

### 2.6. PTPRT/PTPRD Alterations and Progression-Free Survival

**Figure 4:**
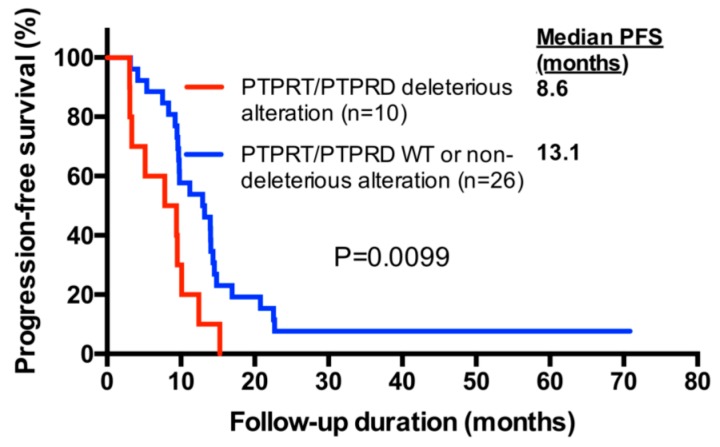
Progression-free survival (PFS) according to *PTPRT/PTPRD* alteration status. Progression-free survival upon bevacizumab treatment of all study cohort patients is shown in a Kaplan–Meier plot. Statistical analysis was performed with the Log-rank test.

We further evaluated progression-free survival (PFS) depending on the presence of postulated deleterious *PTPRT/PTPRD* alterations. Progression-free survival was significantly shorter in bevacizumab-treated patients harboring postulated deleterious *PTPRT/PTPRD* alterations, the median PFS being 8.6 months compared to 13.1 months for patients without deleterious alterations (Hazard Ration (HR), 2.50; 95% CI, 1.40–9.52; *p* = 0.0099, Log-rank test) (Figure 4). Among the patients without deleterious *PTPRT/PTPRD* alterations, the eight non-responders had median PFS of 9.4 months, which was similar to the median PFS of 8.6 months in patients harboring such alterations. In contrast, responding patients, all of whom did not harbor deleterious *PTPRT/PTPRD* alterations, had a median PFS of 14.0 months. The survival in these patients was significantly longer compared to non-responders without deleterious *PTPRT/PTPRD* alterations (HR, 0.39; 95% CI, 0.09–0.79; *p* = 0.0197, Log-rank test).

In addition to *PTPRT/PTPRD* deleterious alterations, univariate Cox regression identified the primary tumor site as a factor associated with PFS (Table 3). In multivariate analysis, both postulated deleterious *PTPRT/PTPRD* alterations and rectal tumors were independent factors associated with shorter PFS (HR, 3.33; 95% CI, 1.47–7.54; *p* = 0.0038 and HR, 2.56; 95% CI, 1.23–5.32; *p* = 0.0118, respectively). However, although associated with PFS, tumor site was not associated with response (*p* = 0.4998, Fisher’s exact test).

## 3. Discussion

In mCRC, bevacizumab has been shown to prolong survival and increase response rates when used in first-line treatment with irinotecan-based regimens [4,5,6,7]. However, although not all patients benefit from bevacizumab, there are no clinically established predictive biomarkers to guide patient selection, according to clinical practice guidelines [8,9] and the European Medicines Agency (EMA) report [10]. Our study is the first to identify the predictive utility of *PTPRD/PTPRT* genetic variants in mCRC patients receiving bevacizumab-based chemotherapy. In our study cohort, the absence of *PTPRD/PTPRT* deleterious genetic variants conferred a statistically significant and clinically meaningful increase in response rate and survival benefit. 

Our results showed that deleterious mutations in *PTPRT*, but not mutations in other genes frequently mutated in our cohort, such as *TP53*, *KRAS*, *APC*, and *SYNE1*, were associated with lack of bevacizumab response (relative risk, 2.39; 95% CI, 1.58–3.61, *p* = 0.0455). Pathway-based analysis revealed that additional consideration of mutations in the *PTPRT*-related gene *PTPRD* involved in JAK/STAT signaling further improved the prediction of bevacizumab response (in total, 38.9% (*n* = 7) of non-responders harbored deleterious *PTPRT/PTPRD* mutations, whereas such mutations were not detected in any responders (relative risk of non-response in patients with deleterious *PTPRT/PTPRD* mutations, 2.64; 95% CI, 1.66–4.20, *p* = 0.0076). In contrast, no associations of deleterious mutations in genes of the RTK/RAS/PI3K pathway, the *Wnt* pathway, and the *TGFβ* pathway with response were found. 

Both *PTPRD* and *PTPRT* are protein tyrosine phosphatases (PTPs). Protein tyrosine phosphatases can act as tumor suppressors which interfere with signaling in malignant cells by counteracting tyrosine kinases [16,17,18]. Among PTPs, *PTPRD* and *PTPRT* belong to the receptor protein tyrosine phosphatases (PTPRs). Their functional relevance is demonstrated by the fact that ectopic *PTPRD* expression induces apoptosis in cancer cells and inhibits their oncogenic, metastatic properties [19,20], and *PTPRT* knockout mice are highly sensitive to carcinogen-induced colon cancer [21].

Among PTPRs, *PTPRT* exhibits the highest mutation prevalence in malignancies. *PTPRT* mutations were identified in various cancer types and are particularly common in CRC [22,23,24]. In our study, we found *PTPRT* to be mutated in approximately 25% of patients. A previous study found a prevalence of 26% for mutations in *PTPRT* and other phosphatases, with a high proportion of *PTPRT* mutations [22]. We postulate five of eight *PTPRT* mutations detected in our cohort to be deleterious, one variant being a truncating variant and the other four being cancer-recurrent missense mutations. Three of those cancer-recurrent missense mutations were detected in the phosphatase domains, and such mutations have been demonstrated to be associated with reduced phosphatase activity [22]. 

*PTPRD* has also been found to be mutated in CRC and in various other cancer types [19,25,26] and is frequently inactivated by copy number loss in a broad range of cancer types [27,28,29]. Similar to TCGA data [30], copy number loss of *PTPRD* was more frequent than that of *PTPRT* in our cohort, in which no *PTPRT* loss was observed. Copy number loss of *PTPRD* was only detected in bevacizumab non-responders. When combining *PTPRT/PTPRD* copy number loss and deleterious mutations, 55.6% of bevacizumab non-responders, but none of the responders harbored deleterious genetic *PTPRT/PTPRD* alterations ((relative risk, 3.25; 95% CI, 1.83–5.79, *p* = 0.0003). Interestingly, deleterious *PTPRT* and *PTPRD* alterations were mutually exclusive in the vast majority of our study patients. This result indicates that the two phosphatases may not be functionally redundant, since the loss of one phosphatase cannot be compensated by the other phosphatase. 

The finding that inactivation of *PTPRT/PTPRD* is associated with bevacizumab resistance may be explained by their role in signal transduction. *PTPRT/PTPRD* dephosphorylate various signaling molecules, stabilize cell-cell adhesions and inhibit tumor migration [21,31,32,33]. One of their target molecules is the signal transducer and activator of transcription factor 3 (STAT3) [34,35]. STAT3 is induced in tumor cells under hypoxic conditions, which triggers tumor cell production of hypoxia inducible factor 1 alpha (HIF-1α) and VEGF; VEGF then acts on endothelial cells to promote cell proliferation and angiogenesis [36]. Additionally, VEGF can stimulate tumor cells in an autocrine manner, and bevacizumab resistance has been associated with upregulation of VEGF and its receptors VEGFR1 and VEGFR2 by tumor cells [37,38]. Therefore, *PTPRT/PTPRD* deleterious alterations may promote bevacizumab resistance by the upregulation of components of the VEGF pathway.

Most biomarker studies have focused on components of the VEGF pathway to predict bevacizumab outcome. However, results have been mostly disappointing. Baseline VEGF-A levels were not associated with bevacizumab response [39,40]. Contradictory results regarding the predictive value of response and/or survival were published for VEGFR1 rs9582036 [13,41] and VEGFA rs833061 [11,13,14,42], although ethnic differences between cohorts may explain part of the observed differences. A comparison of response rates of our study and the abovementioned studies with statistically significant findings demonstrate that in the present study, there is a high difference in response rates between biomarker-positive versus -negative patients (*PTPRT/PTPRD* deleterious alterations vs no deleterious alterations; 0% vs. 69.2%, VEGFR1 rs9582036 CC vs. AA; 36% vs. 56% [41]; VEGFA rs833061 CC vs. TT/TC: 20% vs. 56% [42] and VEGFA rs833061 all genotypes other than T/T vs. T/T; 51% vs. 76% [14]). However, it was suggested that angiogenesis might be too complex for single germline SNPs to serve as reliable predictors of bevacizumab benefit [13], and a combination of biomarkers might improve patient stratification. In this regard, in a very small cohort (*n* = 14) for which a biomarker combination approach was used for VEGF-A/VEGFR1/VEGFR2 mRNA expression, 0% of responders, but 71% of non-responders had a low signature expression [43]. Our approach includes a combination of genetic alterations in two genes, and in the future, it may be interesting to evaluate effects of combinations with other biomarkers.

In agreement with limited response to bevacizumab, patients with *PTPRT/PTPRD* deleterious alterations also had an inferior PFS (8.6 months versus 13.1 months, HR, 2.50; 95% CI, 1.40–9.52, *p* = 0.0099). In multivariate analysis, *PTPRT/PTPRD* deleterious alterations retained their statistical significance as a predictor of short PFS (HR, 3.33; 95% CI, 1.47–7.54; *p* = 0.0038). The other factor associated with short PFS were rectal tumors, although rectal tumors were not associated with the lack of response. The association of rectal tumors with inferior PFS was surprising, considering that PFS in rectal tumor patients treated with bevacizumab and chemotherapy has been reported to be longer than for most other tumor sites [44]. Regarding tumor sites, it has further been reported that splenic flexure tumors have a high proportion of *PTPRD* mutations [45]. Although it is possible that patients with specific tumor locations are more likely to harbor *PTPRD* mutations and could be more likely to be bevacizumab resistant, our cohort only included two patients with splenic flexure tumors, and was therefore not suitable to confirm those results. However, we observed no *PTPRD* mutation in any of those two patients, although one patient harbored a heterozygous *PTPRD* loss. 

The strength of our study is a comprehensive genetic analysis of patient samples without preselecting potential biomarkers. The potential of the identified marker genes to interfere with angiogenesis supports their biological validity. However, the retrospective nature and the small cohort size represent major limitations. Therefore, our results need to be confirmed in larger prospective studies.

## 4. Materials and Methods

### 4.1. Patients

For this study, tumor samples from 36 mCRC patients treated at the Oncology Department of the Chang Gung Memorial Hospital, Tao-Yuan, Taiwan from 2011 to 2015 were analyzed. Study eligibility criteria were the histologically confirmed diagnosis of colorectal adenocarcinoma, the presence of metachronous or synchronous metastases, measurable tumor lesions to evaluate therapeutic responses, ECOG performance status 0–1 and no previous bevacizumab-based therapy. The study received approval from the Chang Gung Memorial Foundation Institutional Review Board (IRB 102-2850A3). All patients provided written informed consent before tumor samples were obtained. Patient characteristics are listed in Table 1.

### 4.2. Treatment Regimens

In the first-line therapy settings, patients with mCRC received bevacizumab plus chemotherapy with FOLFIRI (i.e., irinotecan and infusional 5-fluorouracil with leucovorin) [46]. In the second-line settings, patients with mCRC who had progressed after oxaliplatin-based chemotherapy received bevacizumab plus chemotherapy with FOLFIRI [46]. The detailed chemotherapy and bevacizumab schedules are described as follows: bevacizumab (5 mg per kg of bodyweight) infused initially over 30–90 min on day 1, thereafter with 60–90 min infusion of irinotecan (180 mg per m^2^ of body-surface area); 120 min infusion of folic acid (400 mg per m^2^ of body-surface area) with an intravenous bolus of 5-fluorouracil (400 mg per m^2^ of body-surface area), and then a continuous 46 h infusion of 5-fluorouracil (2400 mg per m^2^ of body-surface area) [47,48]. Response to therapy was evaluated by CT scans using Response Evaluation Criteria in Solid Tumors (RECIST) criteria 1.1. [49].

### 4.3. Tumor Sequencing and Analysis of Genetic Alterations

DNA extraction from formalin-fixed paraffin-embedded (FFPE) tumor samples was performed with the QIAamp DNA FFPE Tissue Kit (Qiagen, Hilden, Germany). For DNA quantification, the Qubit™ dsDNA HS Assay Kit (Invitrogen, Carlsbad, CA, USA) was used. DNA amplification and sequencing were performed with the Ion AmpliSeq Comprehensive Cancer Panel (Life Technologies, Carlsbad, CA, USA) and an Ion Proton sequencer with an Ion P1 chip (Life Technologies), respectively. The mean sequencing depth was >800×. For variant analysis, the human genome sequence hg19, the Torrent Suite Server version 5.0 and the Torrent Suite Variant Caller plug-in, version 5.0 were used. Variants with a frequency of ≥5%were included in the further analysis. In addition to single nucleotide variants and small insertions and deletions, copy number variants were analyzed by ONCOCNV (https://github.com/BoevaLab/ONCOCNV) [50].

Mutations were postulated to be deleterious if they were cancer-recurrent variants and therefore had a corresponding COSMIC ID. Additionally, mutations in TSGs were considered as deleterious if they were frameshift, nonsense or splice-site mutations. For the localization of mutations in *PTPRT/PTPRD* functional domains and their display in protein graphs, tools from cbioportal were used [51,52].

### 4.4. Statistical Analysis

Statistical analysis was performed using GraphPad Prism (v. 6.0; GraphPad Inc, San Diego, CA, USA) and SPSS (v. 20.0.0; IBM, New York, NY, USA). Categorical outcomes were compared using the Fisher’s exact test, whereas PFS was analyzed by the Log-rank test or Cox regression, as indicated. For multivariate Cox regression, all factors with a *p*-value < 0.25 in univariate Cox regression were included.

## 5. Conclusions

The present study demonstrated the clinical utility of performing comprehensive NGS profiling in mCRC patients to identify genetic markers of bevacizumab response. Through integrative analysis of cancer genetic and clinicopathological data, deleterious alterations in *PTPRT/PTPRD* were associated with bevacizumab resistance, as indicated by a poor response rate and shorter survival. This is the first study, to our knowledge, reporting that *PTPRT* and *PTPRD* deleterious alterations predict bevacizumab sensitivity in mCRC. However, the potential of *PTPRT/PTPRD* alterations as predictive markers for bevacizumab therapy in mCRC warrants further confirmation by pre-clinical and clinical studies. 

## Figures and Tables

**Table 1:** Patient characteristics.

	All Patients	Responders	Non-Responders	*p* Value
	*n* (%)	*n* (%)	*n* (%)	
**Total number**	36 (100)	18 (50)	18 (50)	
**Sex**				0.500
**Male**	21 (58.3)	12 (67.7)	9 (50.0)	
**Female**	15 (41.7)	6 (33.3)	9 (50.0)	
**Age**				0.740
**<60**	18 (50.0)	10 (55.6)	8 (44.4)	
**≥60**	18 (50.0)	8 (44.4)	10 (55.6)	
**Median (range)**	59.5 (33–87)	57 (40–84)	61.5 (33–87)	
**Histologic grade**				1.000
**low grade ^1^**	33 (91.7)	16 (88.9)	17 (94.4)	
**high grade ^2^**	3 (8.3)	2 (11.1)	1 (5.6)	
**Metastatic pattern**				1.000
**metachronous**	17 (47.2)	8 (44.4)	9 (50.0)	
**synchronous**	19 (52.8)	10 (55.6)	9 (50.0)	
**Primary tumor site**				0.500
**Colon**	21 (58.3)	12 (66.7)	9 (50.0)	
**Rectum**	15 (41.7)	6 (33.3)	9 (50.0)	
**Metastatic site**				
**Liver**	21 (58.3)	8 (44.4)	13 (72.2)	
**Lung**	15 (41,7)	9 (50.0)	6 (33.3)	
**Other**	15 (41.7)	9 (50.0)	6 (5.6)	
**Number of metastatic sites**				1.000
**1**	21 (58.3)	10 (55.6)	11 (61.1)	
**>1**	15 (41.7)	8 (44.4)	7 (38.9)	
**Treatment regimen**				0.603
**1st line**	32 (88.9)	15 (83.3)	17 (94.4)	
**2nd line**	4 (11.1)	3 (16.7)	1 (5.6)	
**PFS (months)**				0.001
**Median (range)**	9.8 (3.0–70.9)	14.0 (7.5–70.9)	9.2 (3.0–22.5)	

Statistical analysis was performed with the Fisher’s exact test or the Log-rank test, as appropriate. ^1^ Well-differentiated/moderately-differentiated. ^2^ Poorly-differentiated. PFS: progression-free survival.

**Table 2:** List of all *PTPRT* and *PTPRD* sequence variants and copy number losses detected in our study cohort.

Sample ID	Response	Gene	cDNA Change	Position/AA Change	COSMIC ID	Protein Domain	Variant Type	Deleterious
B00524	PD	*PTPRT*	c.3970C>T	p.R1324W	COSM577363	Phosphatase	Missense	Yes
B00524	PD	*PTPRD*					Heterozygous deletion	Yes
B00523	PD	*PTPRT*	c.524delG	p.G175fs			Frameshift	Yes
A00023	PD	*PTPRD*					Heterozygous deletion	Yes
B00547	SD	*PTPRT*	c.2941G>A	p.A981T	COSM1318833	Phosphatase	Missense	Yes
B00550	SD	*PTPRT*	c.3073G>A	p.V1025I	COSM3546449	Phosphatase	Missense	Yes
B00559	SD	*PTPRD*	c.3715-8T>C	Splice region		Splice region	Splice region	Yes
B00561	SD	*PTPRD*					Heterozygous deletion	Yes
B00570	SD	*PTPRD*					Heterozygous deletion	Yes
B00518	SD	*PTPRT*	c.1186G>A	p.V396I	COSM1411875	MAM	Missense	Yes
B00562	SD	*PTPRD*	c.353-4G>C	Splice region		Splice region	Splice region	Yes
B00562	SD	*PTPRD*	c.4855G>A	p.E1619K		Inter-domain	Missense	No
A00074	PR	*PTPRT*	c.774_775 delCCinsAA	p.Q259K		Inter-domain	Missense	No
B00535	PR	*PTPRT*	c.1895G>A	p.R632Q		Inter-domain	Missense	No
B00554	PR	*PTPRT*	c.550G>T	p.V184F		MAM	Missense	No
A00013	PR	*PTPRD*	c.4157A>G	p.Y1386C			Missense	No

MAM: meprin-A5 antigen-PTP; PR: partial response; SD: stable disease; PD: progressive disease; COSMIC: Catalogue of Somatic Mutations in Cancer.

**Table 3:** Uni- and multivariate analysis of progression-free survival.

Factors	*n*	HR ^1^	95% CI ^1^	*p* Value ^1^	HR ^2^	95% CI ^2^	*p* Value ^2^
**Clinical**							
Sex (male/female)	21/15	1.34	0.66–2.69	0.4164			
Age (≥60/<60)	18/18	1.13	0.57–2.25	0.7191			
Histologic Grade (high/low)	3/33	1.61	0.48–5.46	0.4422			
Metastatic pattern (meta/syn)	17/19	1.19	0.60–2.35	0.6144			
Primary Tumor site (rectum/colon)	15/21	2.11	1.04–4.26	0.0377	2.56	1.23–5.32	0.0118
Number of metastatic sites (>1/1)	15/21	1.01	0.51–2.01	0.9757			
Treatment regimen (2nd line/1st line)	4/32	0.77	0.27–2.20	0.6274			
**Genetic**							
*PTPRT/PTPRD* deleterious alteration (yes/no)	10/26	2.67	1.23–5.80	0.0130	3.33	1.47–7.54	0.0038

HR: hazard ratio. ^1^ Univariate analysis. ^2^ Multivariate analysis.

## Supplementary Materials

The following are available online at http://www.mdpi.com/2072-6694/10/9/314/s1, Table S1: list of genes assayed, Table S2: complete list of all sequence variants detected in the study cohort, Table S3: association of postulated deleterious mutations in frequently mutated genes and key signaling pathway genes with bevacizumab response status. (statistical analysis was performed with the Fisher’s exact test).
