# Supplementary material for: PTPRT and PTPRD Deleterious Mutations and Deletion Predict Bevacizumab Resistance in Metastatic Colorectal Cancer Patients
Source: Cancers (Basel). 2018 Sep 6;10(9):314. doi: 10.3390/cancers10090314 (PMC6162606; doi:10.3390/cancers10090314)
Supplement: Supplementary file 1 [file cancers-10-00314-s001.zip › cancers-335170-sup/Supplementary Table S3.docx]

**Supplementary Table S3. Association of postulated deleterious mutations in frequently mutated genes and key signaling pathway genes with bevacizumab response status.**

|  | **Pathway/ genes** | **Status** | **Treatment outcome** | | ***p* value** |
| --- | --- | --- | --- | --- | --- |
|  |  |  | **Responder (n=18)** | **Non-responder (n=18)** |  |
| **Frequently mutated gene** | ***TP53*** | **WT or non-del mut** | **4** | **4** | **1.0000** |
|  |  | **Del mut** | **14** | **14** |  |
|  | ***KRAS*** | **WT or non-del mut** | **6** | **5** | **1.0000** |
|  |  | **Del mut** | **12** | **13** |  |
|  | ***APC*** | **WT or non-del mut** | **7** | **8** | **1.0000** |
|  |  | **Del mut** | **11** | **10** |  |
|  | ***SYNE1*** | **WT or non-del mut** | **18** | **17** | **1.0000** |
|  |  | **Del mut** | **0** | **1** |  |
|  | ***PTPRT*** | **WT or non-del mut** | **18** | **13** | **0.0455** |
|  |  | **Del mut** | **0** | **5** |  |
| **Pathway** | **RTK/PI3K/RAS *(BRAF, ERBB2, HRAS*, KRAS, NF1*, NRAS*, PIK3CA, PTEN*)*** | **WT or non-del mut** | **3** | **5** | **0.6906** |
|  |  | **Del mut** | **15** | **13** |  |
|  | **Wnt *(APC, CTNNB1*)*** | **WT or non-del mut** | **7** | **8** | **1.0000** |
|  |  | **Del mut** | **11** | **10** |  |
|  | **TGFβ *(SMAD2, SMAD4)*** | **WT or non-del mut** | **15** | **15** | **1.0000** |
|  |  | **Del mut** | **3** | **3** |  |
|  | **JAK/STAT *(JAK1, JAK2*, JAK3, PTPRD, PTPRT)*** | **WT or non-del mut** | **18** | **11** | **0.0076** |
|  |  | **Del mut** | **0** | **7** |  |

Statistical analysis was performed with the Fisher’s exact test.
